# Effect of thoracic mobilization exercises on hamstring flexibility: a randomized controlled trial

**DOI:** 10.55730/1300-0144.5695

**Published:** 2023-06-12

**Authors:** Aybüke ERSİN, Meltem KAYA

**Affiliations:** Department of Physiotherapy and Rehabilitation, Faculty of Health Sciences, İstanbul Atlas University, İstanbul, Turkiye

**Keywords:** Hamstring muscles, exercise, pain, fascia

## Abstract

**Background and aim:**

The aim was to investigate the effect of thoracic mobilization exercises on hamstring flexibility.

**Materials and methods:**

One hundred twenty individuals with hamstring tightness were included in the study. The participants were randomized into two groups: the experimental group (EG) (n = 60) or the control group (CG) (n = 60). The EG performed a home-based thoracic mobilization exercise program comprising 2 sets with 10 repetitions, once a day, 3 days a week, for 4 weeks. The CG performed active-assisted stretching of the hamstring comprising 3 sets with 10 repetitions for 15 seconds. The active knee extension (AKE) test was used to measure hamstring flexibility, and self-reported hamstring pain intensity was evaluated with a visual analog scale (VAS). All evaluations were conducted at the beginning and end of the intervention.

**Results:**

Both groups showed significant improvement in AKE (p<0.05). Pain intensity during the stretching exercises was significantly decreased only in the EG. The improvements in AKE and VAS score were greater in the EG than in the CG (p < 0.05). Between-group effect sizes were large for AKE (d = 1.075) and VAS score (d = 1.077).

**Conclusion:**

The current study showed that thoracic mobilization exercises may increase hamstring flexibility and reduce pain intensity during hamstring stretch exercises.

## 1. Introduction

The muscle–tendon complex plays a crucial role in enhancing both the effectiveness and efficiency of body movements. Flexibility is a fundamental property of reducing the risk of injury and muscle soreness and maintaining normal biomechanical functions [1,2]. The hamstring is a muscle group that commonly has reduced flexibility and causes lower extremity injuries including strain, lower and upper back pain, patellar tendinopathy, and patella femoral pain [3 4].

The classic perspective describes the hamstring as being isolated from the adjacent structures. However, this concept has recently been revised, i.e., decreased flexibility is due to adaptive shortening of the musculature, tendons, and other soft tissues especially such as fascia [5]. A considerable amount of literature has been published on the fascia link between the active components of the movement system to form a network of meridians or myofascial chains [6,7]. The superficial posterior line, which is one of these meridian systems, connects the hamstring with the thoracolumbar fasciae, the erector spinae, and the epicranial aponeurosis cranially. Due to the location of the hamstring with its origin at the ischial tuberosity, dysfunction in any of these systems may also indirectly affect hamstring flexibility [8,9].

Various treatment techniques are currently used to improve hamstring flexibility; one of the most widely recommended is stretching, including static, dynamic, active-self, and ballistic stretching and proprioceptive neuromuscular facilitation [10]. Despite these clinically and experimentally successful methods, no consensus has been reached on a standard protocol for treatment. Thus, there has been increasing interest in active exercise interventions with more attention given to possible disturbances within the functional anatomical interaction and the kinetic chain between the hamstring and columna vertebralis [11–13].

Spinal mobilization exercises have been reported to provide both neurophysiological and biomechanical benefits [14]. In previous studies thoracic mobilization exercises were seen to improve range of motion, pain, lumbosacral alignment, and functional status [15–17]. Although there is functional and anatomical evidence of a fascial link between the hamstring and thoracic spine, there remains a lack of research on the effect of thoracic mobilization exercises on hamstring flexibility.

The aim of the present study was to investigate the effectiveness of thoracic mobilization exercises on hamstring flexibility in healthy adults. It is hypothesized that thoracic mobilization exercises will significantly increase hamstring flexibility and decrease self-reported hamstring pain intensity.

## 2. Materials and methods

### 2.1. Study design and participants

The study was conducted as a prospective and randomized controlled study. To investigate the effect of thoracic mobilization exercises on hamstring flexibility and pain during stretching exercises 120 healthy subjects of either sex between the ages of 18 and 45 years were recruited. The inclusion criteria were as follows: willingness to participate in the study, bilateral hamstring tightness, and ability to read written and understand spoken language. Hamstring tightness was defined as less than 70° hip flexion angle on the passive straight leg raising test [18]. Those with knee or low back pain, history of fractures of the lower limbs, muscle/tendon lesions of the hamstring, plantar fasciitis, and recent foot and ankle injuries within the previous 1 year and those unable to perform tests or exercises because of diagnosed comorbidities were excluded from the study.

The study was approved by the Noninterventional Scientific Research Ethics Committee of Istanbul Atlas University (Protocol number: E-22686390-050.99-18385) and performed in accordance with the Declaration of Helsinki. Written informed consent was obtained from all participants. The study was completed without any participant dropout.

### 2.2. Outcome measurements

All initial and final assessments were performed by the same physiotherapist. The sociodemographic and anthropometric parameters of the subjects (age, sex, height, weight, and body mass index) were recorded. Knee extension range of motion was measured to determine hamstring flexibility and pain intensity during hamstring stretching exercises.

To measure hamstring flexibility, the active knee extension (AKE) test was applied to the dominant leg. The participants lay in the supine position with the tested hip flexed 90° and knee flexed 90° and were asked to extend the knee as much as possible until they felt strong resistance. Complete knee extension was 0° and lack of knee extension was measured with a goniometer [19]. Excellent interrater (ICC: 0.87) and intrarater reliability (ICC: 0.97) for assessing hamstring flexibility with the AKE test, which is performed easily by a single assessor, was seen [20].

Pain intensity during hamstring stretch exercises was measured by visual analog scale (VAS). The VAS is a graphic tool with a 100-mm horizontal line with the left end marked with “no pain” and the right end marked with “worst imaginable pain”. The participants were asked to draw a vertical mark on the line to represent their level of pain intensity. The length from the left end to the vertical mark made by the participant was measured in millimeters [21]. In the supine position, hamstring stretching using a stretching cord was used to evaluate worst pain during stretching by staying 30 s in the stretched position. The VAS is a simple and reliable instrument for assessing muscoskeletal pain intensity in clinical settings and research (ICC: 0.82) [22].

After the initial assessments, the patients were randomized into two groups: the experimental group (EG) (n = 60) or the control group (CG) (n = 60) using computer software.

### 2.3. Interventions

The participants in the EG performed a home-based thoracic mobilization exercise program. The exercises comprised 2 sets with 10 repetitions, once a day, 3 days a week, for 4 weeks. The exercise programs consisted of the cat/camel exercise, thoracic extension at a wall using bodyweight, half-kneeling chop/thrust, and side-lying thoracic rotation (Figure 1). The exercises are designed to improve thoracic spine mobility, spinal alignment, and muscular strength. For the cat/camel exercise (Figure 1a), the participants were in the prone kneeling position and took a deep breath through the nose while arching the back (cat) and breathed out through the mouth while rounding the spine (camel). The thoracic extension exercise (Figure 1b) was performed while standing facing a wall. The participants walked back and moved the hands down the wall until their chest was parallel to the ground. The half-kneeling chop/thrust exercise (Figure 1c) was applied in a half-kneeling position. The movement was started by rotating the trunk to the contralateral side of the foreleg, with the chest facing in a direction roughly perpendicular to that of the foreleg. The arms were lifted overhead while doing so. The motion was completed by rotating the torso towards the opposite side of the body while bringing the arms down in the same direction. Lastly, the side-lying thoracic rotation exercise (Figure 1d) was started lying on the side with the legs flexed at 90° and the arms together straight in front of the participants. The top arm was moved slowly away from the other arm, toward the floor on the other side, rotating the trunk at the same time.

**Figure 2:**
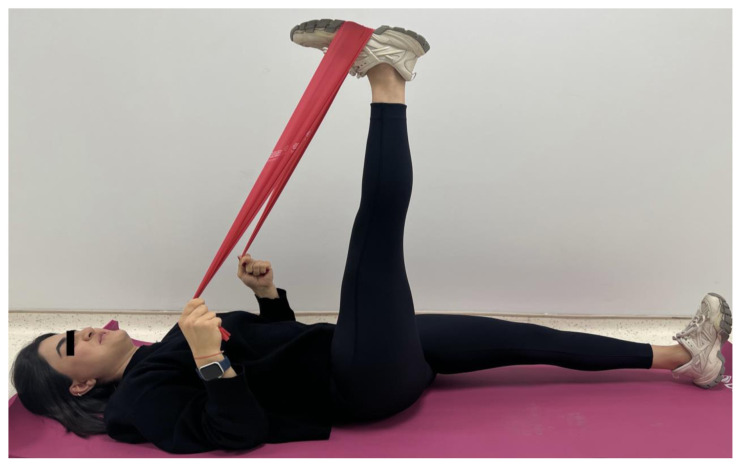
Hamstring stretching (control group).

The CG performed active-assisted stretching of the hamstring in the supine position using an exercise band. The exercise consisted of increasing hip flexion while the knee was extended until they felt tightness and they stayed in this position for 15 s keeping the back straight. The stretching was done in 3 sets of 10 repetitions with a 15-s rest interval between each stretch (Figure 2).

All evaluations were repeated at the end of 4 weeks. The participants were requested to keep an exercise diary. Adherence to the exercise program (%) was defined as the ratio of the completed sessions to total sessions, which was calculated as follows: (Completed sessions)/(Total sessions = 12 sessions) multiplied by 100.

### 2.4. Statistical analysis and sample size

SPSS v.26 (SPSS Inc., USA) was used for the data analysis. The normality of the distribution of data was analyzed using the Shapiro–Wilk test. Categorical variables were compared between the groups using the chi-squared test. The paired samples t-test or independent samples t-test was used for intragroup comparisons and the Wilcoxon or Mann–Whitney U test was used for between-group comparisons depending on the distribution properties of the data. The statistical significance level was set at p < 0.05 for all analyses. The effect size (Cohen’s d) was calculated, and Cohen’s d results represent 0.8 large, 0.5 medium, and 0.2 small effect [23].

Sample size was calculated using G*Power 3.1 (Universität Düsseldorf, Germany) [24]. The calculation was based on previous work by Aparicio [25], who recorded hamstring flexibility changes using an experimental and a control group. Based on their hamstring flexibility intragroup changes, power analysis for the current study indicated that a total of 120 subjects (60 per group) would enable a difference in hamstring flexibility (as measured by AKE test) to be detected with 0.518 effect size at a 5% significance level with 80% power.

## 3. Results

**Figure 3:**
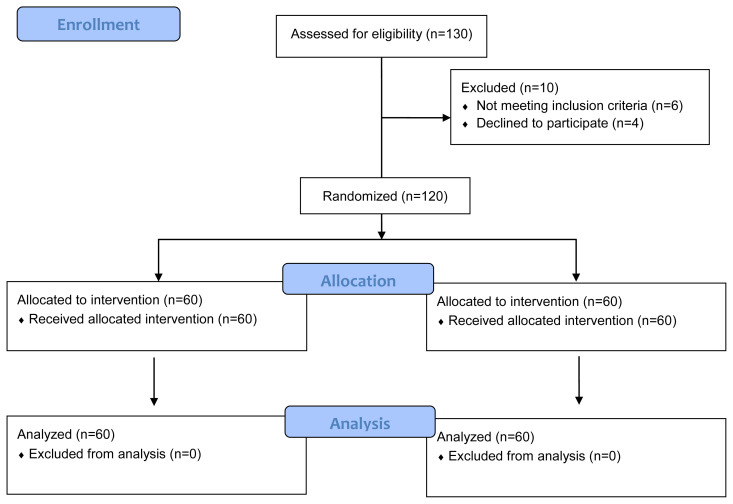
CONSORT flow diagram.

One hundred thirty volunteers were assessed for eligibility and 10 were excluded for not meeting the inclusion criteria or refusing to participate. Sixty participants for each group were included in the study and a total of 120 completed the study with no dropouts (Figure 3). The demographic and anthropometric parameters of the participants are shown in Table 1. No significant difference was found between the two groups’ demographic and anthropometric data. Adherence to the exercise program (%) was 89.10 ± 13.81 in the EG and 88.10 ± 13.21 in the CG with no between-group difference (p = 0.345).

Table 2 summarizes the effects of the different exercise programs on each outcome. The groups were similar in terms of baseline values (p > 0.05). Both groups showed significant improvement in AKE (p < 0.05). Pain intensity during the stretching exercises was significantly decreased only in the EG. The improvements in AKE and VAS score were greater in the EG than in the CG (p < 0.05). Between-group effect sizes were large for AKE (d = 1.075) and VAS score (d = 1.077).

## 4. Discussion

The aim of the present study was to examine the effect of thoracic mobilization exercises on hamstring flexibility. The improvement in hamstring flexibility was greater in the EG group than in the CG group. Moreover, self-reported pain intensity during hamstring stretching was reduced only in the EG group.

The superficial back line (SBL) connects the hamstring with the gastrocnemius muscles and the plantar fascia caudally and with the thoracolumbar fasciae, the erector spinae muscle, and the epicranial aponeurosis cranially [26]. In the literature, studies show that different fascia release methods as well as hamstring stretching exercises can increase hamstring flexibility. For example, Fauris et al. showed that the self-myofascial release (MFR) technique they applied to all segments increased hamstring flexibility [26]. Gyanpuri et al. reported that both the MFR technique applied to the plantar fascia and the postisometric relaxation technique increase hamstring flexibility, but argue that the MFR technique is more effective [27]. In addition, Borsaniya reported that the kinetic chain activation technique, which reduces tension in the hamstring fascia, improves hamstring flexibility [28]. Our results show that thoracic mobilization exercises increase hamstring flexibility. This increase can be explained by the transitions in the thoracolumbal fascia and its relationship with the hamstring. Although there has been little discussion about the minimum clinically important difference of the AKE test, in a previous study it was reported that an improvement of 10.2° could be considered clinically significant [29]. In the present study, in which thoracic mobilization exercises were applied as a flexibility training method, a similar effective result was achieved and an increase of 10.32° was obtained. In the CG, the improvements in the AKE test were statistically significant but not clinically relevant.

When the literature is examined in terms of the techniques used, it is seen that fascial release techniques such as self-myofascial release [30] and kinetic chain activation [28] are more effective in increasing hamstring flexibility than conventional hamstring stretching. Although there are few studies showing the effect of thoracic mobilization or fascial release on flexibility, a considerable number of studies have been published on the effects of static stretching on hamstring flexibility. Worrell et al. [31] and Sullivan et al. [32] reported an 8.0° and 9.2° increase for static stretching in the AKE test. In the present study, the increase in the AKE test was higher in the exercise group. This result may be explained by the fact that fascial activation causes tension and ultimately reduces the overall functioning of the body movements.

Flexibility is an unrestricted pain-free range of motion [28]. In our study, a statistically significant decrease in pain intensity was obtained during hamstring stretching after the thoracic mobilization exercise program. We speculate that the significant decrease in this decrease only in the EG group is related to the contribution of thoracic mobilization exercises to relaxation of the SBL. Aparicio et al. investigated the immediate effect of suboccipital muscle inhibition on hamstring flexibility and pain threshold, and they found that the technique increased hamstring flexibility and the semimembranosus pain threshold [25].

There is clinical evidence that flexibility training may help control the intensity of pain during stretching, especially in tight muscular conditions [33,34]. A strong relationship between the extensibility of the hamstring and stretch tolerance is commonly reported in the literature [35,36]. This result may be explained by the kinetic chain theory. A possible explanation for this is that myofascial tissue meridians can transfer the stress to a distant structure diminishing the related stress and associated pain [6]. Researchers have highlighted that in exercises that include movement of multiple segments in the body, kinetic chain activation has been shown to increase [2]. The present findings appear to be consistent with those of other research. The CG did not show significant improvement in pain intensity during stretching. There is conflicting evidence about the effect of passive stretching training on pain control.

Previous studies have focused on myofascial release techniques to increase hamstring flexibility. To the best of our knowledge, the effect of thoracic mobilization exercises on hamstring flexibility has not been evaluated in any study.

## 5. Limitations of the study

Firstly, the findings of this study cannot be generalized to adolescents and other athletes, especially since they were obtained from healthy recreational male individuals. Secondly, the follow-up period of our study was limited to 4 weeks. It should be investigated whether the gains obtained with the given exercise programs still provide advantages over each other and the control group in the long term. Thirdly, there was no self-release group.

## 6. Conclusion

Thoracic mobilization exercises can be considered as an option during treatment to increase hamstring flexibility as they can be performed at home and do not require any materials.

## Figures and Tables

**Figure 1a:**
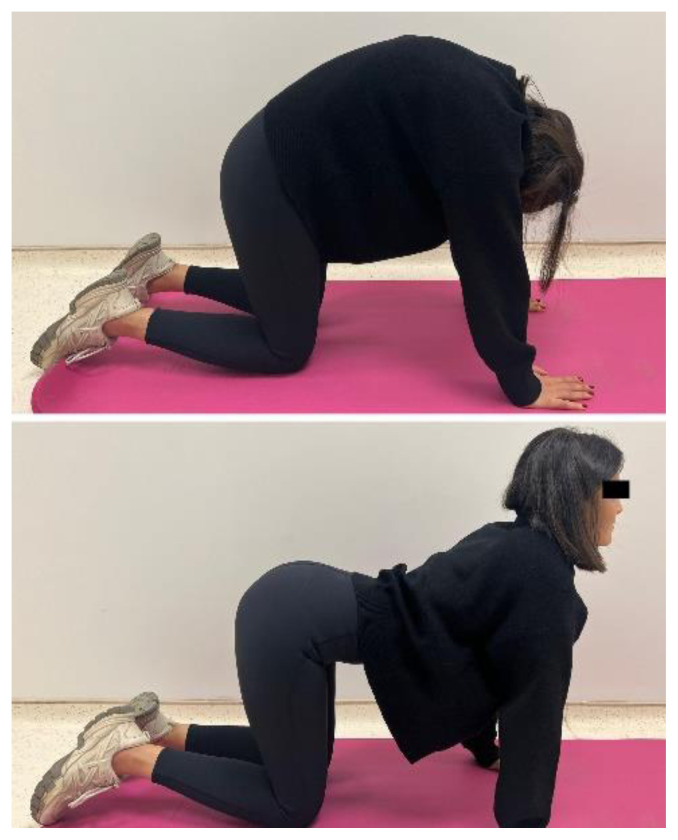
Thoracic mobilization exercises (experimental group).

**Figure 1b:**
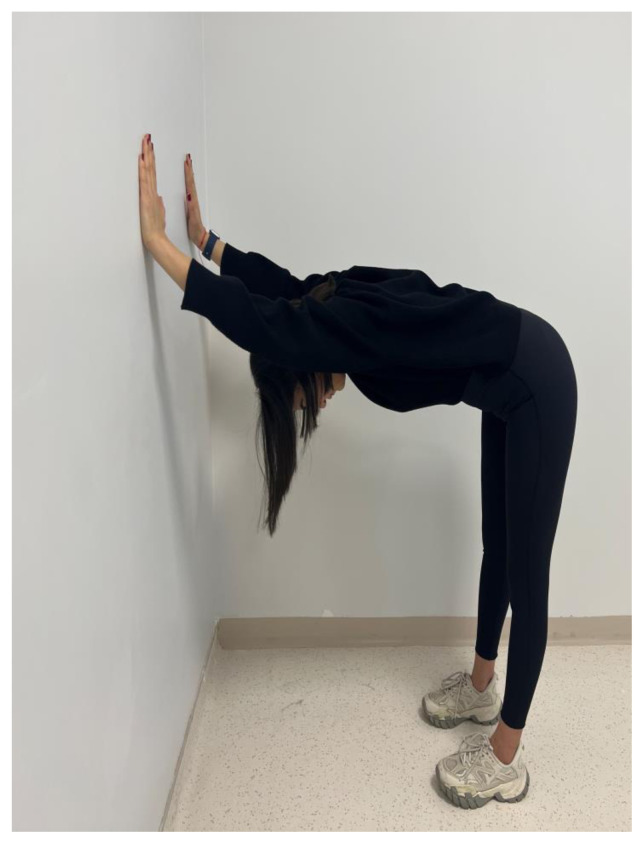
Thoracic mobilization exercises (experimental group).

**Figure 1c:**
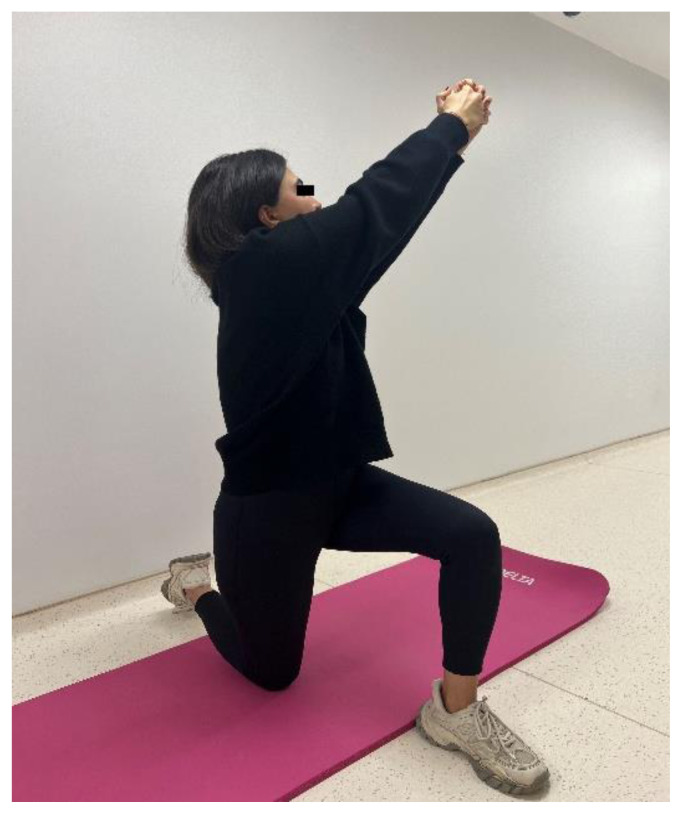
Thoracic mobilization exercises (experimental group).

**Figure 1d:**
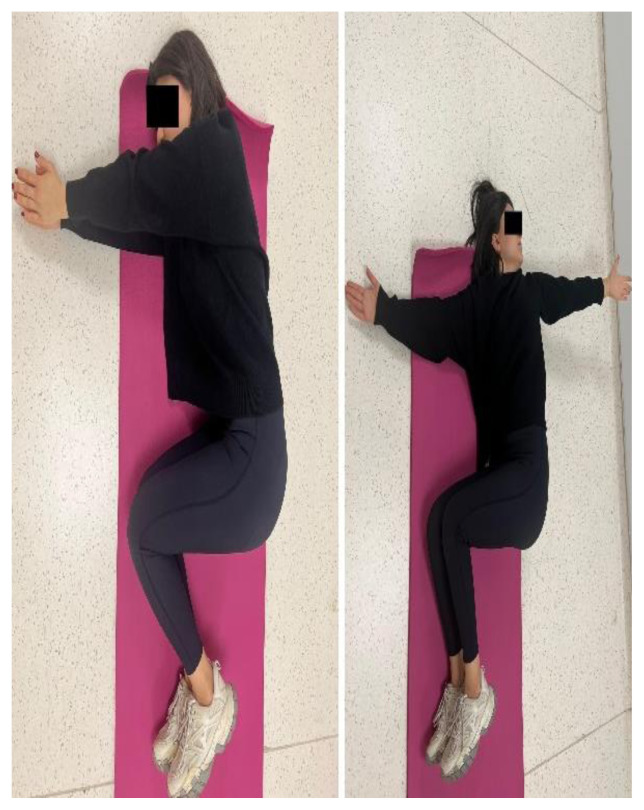
Thoracic mobilization exercises (experimental group).

**Table 1:** Demographic and anthropometric data of the participants.

	EG (n = 60)	CG (n =6 0)	p
**Age (years)**	26.10 ± 5.81	25.52 ± 5.07	0.855†
**Sex**			
Female	40 (66.7%)	43 (38.3%)	0.145‡
Male	20 (33.3%)	17 (61.7%)	
**Body composition** Height (cm)	171.52 ± 8.77	176 ± 10.51	0.431†
Weight (kg)	67.28 ± 14.08	73.85 ± 17.40	0.249†
BMI (kg/m^2^)	23.04 ± 3.73	24.12 ± 4.26	0.245†
**Adherence to exercise program**	89.10 ± 13.81%	88.10 ± 13.21%	0.345†

Data are presented as mean ± standard deviation or n (%). cm: centimeters; kg: kilograms; BMI: Body mass index.

† Independent samples t-test,

‡ Chi-squared test.

**Table 2:** Comparison of the experimental and control groups’ results.

Outcomes	EG (n = 60)	CG (n = 60)	Between-group change	Effect size (Cohen’s d)
**Active knee extension test (degrees)**				
Baseline	27.33 ± 8.86	29.32 ± 7.72		
Postintervention	17.01 ± 7.81	22.93 ± 8.76		
Within-group change	10.32 ± 3.49^a^‡	6.39 ± 3.81^a^‡	3.93 ± 1.69^a^†	1.075
**VAS score (0–100 mm)**				
Baseline	43.33 ± 22.26	50.06 ± 26.55		
Postintervention	30.22 ± 11.45	43.45 ± 15.87		
Within-group change	13.11 ± 5.43^a^‡	6.61 ± 6.58‡	6.5 ± 2.09^a^†	1.077

Data are reported as mean ± standard deviation. VAS: visual analog scale; mm: millimeters.

a Statistically significant differences (p < 0.05).

‡ Paired samples t-test,

† Independent samples t-test.
